# Improving dual protection among women infected with HIV

**DOI:** 10.1186/1471-2334-14-S3-E40

**Published:** 2014-05-27

**Authors:** Beena Joshi, Gajanan Velhal, Sanjay Chauhan, Ragini Kulkarni, Shahina Begum, YS Nandanwar, Michelle Fonseca, Sujata Baweja, Dilip Turbadkar, Anita Ramachandran, Asha Dalal, Jayanti Shastri, Sachee Agrawal, Manisha Panhale, Vasundhara More, Pravin Sanap, Renuka Panchal, Suman Kanoujiya

**Affiliations:** 1 Department of Operational Research, National Institute for Research in Reproductive Health, ICMR, Parel, Mumbai, India; 2 Department of PSM, TN Medical College and BYL Nair Hospital, Mumbai, India; 3 Department of OBGYN, LTM Medical College and Sion Hospital, Mumbai, India; 4 Department of Microbiology, LTM Medical College and Sion Hospital, Mumbai, India; 5 Department of OBGYN, TN Medical College and BYL Nair Hospital, Mumbai, India; 6 Department of Microbiology, TN Medical College and BYL Nair Hospital, Mumbai, India

## Background

The PPTCT program in India focuses on prong 3 (Provision of Nevirapine to pregnant infected mothers) and reports quote that it reaches only 32% of pregnant mothers who need it. Preventing unintended pregnancies among HIV positive women (Prong 2) could help reduce the burden on Prong 3. To improve use of dual protection and prevent unintended pregnancies among women infected with HIV, an operational research study was implemented in two randomly selected tertiary hospitals in Mumbai (supported by ICMR).

## Methods

The main intervention in this experimental control study was linking ICTC/PPTCT with family planning services by capacity building of providers (counseling and testing medical eligibility to use methods), provision of IEC material, referral slip, maintaining MIS and testing the acceptability of dual methods among 300 women infected with HIV attending ICTC/PPTCT.

## Results

Sixty percent of the referred participants reached Family planning centres. Statistically significant improvement in knowledge about contraception and dual protection, three times increased acceptance of dual methods, significant increase in consistent use of condoms and lesser numbers of unwanted pregnancies and births were observed in the experimental than control group. The cumulative failure rate of contraception in the experimental group reduced resulting in preventing 12 women from risk of getting pregnant in first year of use compared to control.

## Conclusion

The study demonstrated the feasibility to establish this linkage and improved dual method use among participants. The study recommends expanding the current mandate of PPTCT to focus on prong 2 and improve use of dual methods.

